# Efficacy and *in vitro* activity of gepotidacin against bacterial uropathogens, including subsets with molecularly characterized resistance mechanisms and genotypes/epidemiological clones, in females with uncomplicated urinary tract infections: results from two global, pivotal, phase 3 trials (EAGLE-2 and EAGLE-3)

**DOI:** 10.1128/aac.01639-24

**Published:** 2025-09-09

**Authors:** Nicole E. Scangarella-Oman, Deborah L. Butler, John Breton, Derrek Brown, Cara Kasapidis, Amanda J. Sheets

**Affiliations:** 1 GSK 525885 , Collegeville, Pennsylvania, USA; University of Pittsburgh School of Medicine, Pittsburgh, Pennsylvania, USA

**Keywords:** gepotidacin, uncomplicated urinary tract infection, acute cystitis, uropathogen, genotype, molecular characterization, antibacterial resistance, nitrofurantoin, extended-spectrum β-lactamase

## Abstract

Gepotidacin, a novel, bactericidal, first-in-class triazaacenaphthylene antibacterial, was noninferior to nitrofurantoin in two pivotal trials (EAGLE-2 and EAGLE-3) in females with uncomplicated urinary tract infections (uUTIs). Using pooled data, gepotidacin *in vitro* activity and clinical efficacy were evaluated for subsets of molecularly characterized isolates in the microbiological Intent-to-Treat population. The subsets of isolates were characterized based on phenotypic/MIC criteria; all microbiological failure isolates were also characterized. Of 1,159 *Escherichia coli* isolates*,* 30% harbored quinolone resistance-determining region (QRDR) mutations; most prevalent was *gyrA* S83L, D87N (27%). Extended-spectrum β-lactamases (ESBLs) were detected in 13% of *E. coli* isolates. For 114 *Klebsiella pneumoniae* isolates, 22% were plasmid-mediated quinolone resistance (PMQR) gene-positive, 11% had QRDR mutations, and 12% had ESBLs. Among 67 *Proteus mirabilis* isolates, 21% harbored QRDR mutations. Gepotidacin MIC_90_ values ranged from 1 to 32 µg/mL against qualifying Enterobacterales uropathogens and genotypic subcategories, with no isolates considered resistant to gepotidacin. For all genotypic subcategories, gepotidacin MIC_90_ values were similar (i.e., lower, equal to, or 1-dilution higher) compared with the overall species (4 µg/mL), with the exception of *E. coli* isolates with the PMQR *qnrS1* gene (16 µg/mL); all were gepotidacin-susceptible. For the majority of uropathogens, including those with genotypes likely to cause resistance to standard uUTI therapies, success rates for gepotidacin were similar across genotypic subcategories for each species. These data show gepotidacin’s efficacy and *in vitro* activity against a wide range of uropathogen genotypes. Additionally, these pooled results provide a robust, contemporary data set and insight into current genotypic mechanisms of resistance.

**CLINICAL TRIALS:**

This study is registered with ClinicalTrials.gov as NCT04020341 and NCT04187144.

## INTRODUCTION

Uncomplicated urinary tract infections (uUTIs) have become more difficult to treat due to increased antibiotic resistance. Empiric oral antibiotic treatment is the standard for uUTIs; however, if treatment is unsuccessful, the risk of progression to a complicated infection more than doubles, leading to both clinical and economic burdens (1). Treatment guidelines attempt to account for antibacterial resistance to identify appropriate antibiotic therapy based on clinical presentation, patient-specific factors, and local surveillance data (2–4), but not all uUTI antibiotic treatment is successful (1, 5).

*Escherichia coli* is the prominent uropathogen in uUTIs and is responsible for approximately 75% of infections (6). *E. coli* sequence type 131 (ST131), a globally disseminated multidrug-resistant clone, is the most prevalent clonal group and has been identified in up to 30% of clinical isolates from various infections, including urine specimens (7). Extended-spectrum β-lactamase (ESBL)-producing *E. coli* ST131 has been recovered in 21% to 34% of isolates from community-acquired urinary tract infections (UTIs) in France, Mexico, and Sweden (8). Bacteria such as *Staphylococcus saprophyticus*, *Proteus* spp., *Klebsiella* spp., and other Enterobacterales are also causative pathogens in uUTIs (6).

Antibiotic resistance due to ESBLs is prevalent in the Enterobacterales order. ESBLs are enzymes capable of inactivating a broad range of penicillins and cephalosporins, including those with an extended spectrum of activity. ESBLs are often encoded on plasmids, which is of concern because these plasmids often carry other resistance genes. These additional resistance genes can confer resistance to other classes of antibiotics, such as aminoglycosides, sulfonamides, and quinolones. This combination of mechanisms results in multidrug resistance, limiting the effectiveness of multiple antibiotic classes in treating UTIs caused by these bacteria (6, 9–12). Given the increasing prevalence of antibiotic resistance due to ESBLs, other genotypes that also lead to antibiotic resistance, and evolving bacterial resistance mechanisms, there is an ongoing need for novel class antibiotic treatment options for uUTIs. This unmet need is especially relevant for oral antibiotics with activity for drug-resistant uropathogens.

Gepotidacin is a novel, bactericidal, first-in-class triazaacenaphthylene antibacterial that inhibits bacterial DNA replication by a distinct binding site, a unique mechanism of action (13, 14), and provides well-balanced inhibition of two different type II topoisomerase enzymes (for most uUTI uropathogens) (15, 16). This provides activity against most strains of uropathogens, such as *E. coli* and *S. saprophyticus*, including isolates resistant to current antibacterials (17, 18). Due to the well-balanced binding at both enzymes (i.e., DNA gyrase and topoisomerase IV), gepotidacin target-specific mutations in both enzymes are needed to significantly affect susceptibility to gepotidacin (15). Therefore, these mechanisms lead to a lower potential for resistance development.

Oral gepotidacin was evaluated in two global phase 3 clinical trials versus the first-line antibiotic, nitrofurantoin, in the treatment of uUTI in female participants (EAGLE-2 [204989] and EAGLE-3 [212390]) (2–4, 19). Results demonstrated statistical noninferiority of gepotidacin (1,500 mg twice daily for 5 days) compared with nitrofurantoin (100 mg twice daily for 5 days) for the composite endpoint of therapeutic response (i.e., combined clinical and microbiological response) at test-of-cure (TOC) in both trials; additionally, statistical superiority of gepotidacin was achieved in EAGLE-3. Based on the results of EAGLE-2 and EAGLE-3 (19), gepotidacin was approved in March 2025 by the US Food and Drug Administration (FDA) for the treatment of female adult and pediatric patients 12 years of age and older weighing at least 40 kg with uUTIs caused by the following susceptible microorganisms: *E. coli*, *K. pneumoniae*, *Citrobacter freundii* complex, *S. saprophyticus*, and *Enterococcus faecalis* (20).

Therapeutic success in these two uUTI trials was contingent on achieving both clinical success and microbiological success. The clinical and microbiological responses were not always concordant. The majority of participants who did not achieve therapeutic success were those who failed in one of the two components (clinical or microbiological), rather than failing in both. In addition, missing or unable to determine results were classified as failures. These stringent success criteria led to lower than anticipated therapeutic success rates for both treatment groups; however, gepotidacin independently showed efficacy in both trials. Acceptable safety and tolerability profiles for gepotidacin were observed in both trials (19).

The EAGLE-2 and EAGLE-3 primary efficacy results were based on the population of participants who had baseline qualifying uropathogen(s) that were required to be susceptible to the active comparator, nitrofurantoin (19). However, the results presented herein are primarily focused on the population of participants who had baseline qualifying uropathogen(s) irrespective of their susceptibility to nitrofurantoin, as well as pooled data across trials, which enlarges the population and data set for analysis.

In a co-publication, the overall distribution of bacterial species recovered at baseline, frequency of drug-resistant phenotypes observed, and gepotidacin activity and efficacy against the phenotypes have been described (21). Here, genotypic data from a subset of baseline qualifying uropathogens and gepotidacin activity and efficacy results for the genotypic subcategories are presented.

In the EAGLE-2 and EAGLE-3 trials (19), all enrolled female participants were required to provide a urine specimen at baseline (prior to treatment) for culture, irrespective of reported UTI symptoms and medical history, including prior antibiotic exposure and history of recurrent UTIs (i.e., unbiased urine collection and culture). A subset of the recovered uropathogen isolates underwent molecular characterization based on phenotypic/MIC criteria and/or association with microbiological failure per a predefined algorithm (Supplemental material). These genotypic data from recently conducted (2019 to 2022) global trials provide a comprehensive and contemporary uropathogen data set.

## RESULTS

### Genotypic subcategories

In the pooled microbiological Intent-to-Treat (micro-ITT) population and combined treatment groups, 30% of the 1,159 baseline qualifying *E. coli* isolates harbored quinolone resistance-determining region (QRDR) *gyrA* mutations with the double S83L, D87N mutation as the most prevalent subcategory (27%) (Table 1). *ParC* mutations were observed in 28% of *E. coli* isolates, with *parC* S80I mutations observed in 16% of *E. coli* isolates. *ParE* L416F mutations were identified in 8% of the baseline *E. coli* isolates characterized. Additionally, plasmid-mediated quinolone resistance (PMQR) genes were detected in 8% of *E. coli* isolates. Sixteen percent of the *E. coli* isolates were β-lactamase gene-positive with ESBLs (13%) as the most prevalent subcategory. Of these ESBLs, CTX-M-15, CTX-M-27, and CTX-M-15, OXA-1_OXA-30 genes were the most common. Extended-spectrum AmpCs and narrow-spectrum *β-*lactamase genotypes were also detected in *E. coli* isolates at frequencies of 9% and 6%, respectively. Only one baseline qualifying *E. coli* isolate had a carbapenemase (OXA-181) (Table S1, which presents all genotypes detected). Among *E. coli* isolates recovered in these trials that met the criteria for multilocus sequence type (MLST) testing and O:H serotyping (i.e., predefined phenotypic/MIC criteria [Supplemental material] plus all microbiological failure isolates), the most common MLSTs and epidemiological clones identified were ST131, ST1193, and O25b:H4; however, as only a subset of isolates were tested, the data do not reflect the overall prevalence among *E. coli. FimH*30 was the most common *fimH* allele among the *E. coli* ST131 isolates tested.

**TABLE 1:** Incidence of selected baseline qualifying uropathogens recovered and genotypes/epidemiological clones with *n* ≥ 10 isolates across treatment groups for pooled EAGLE-2 and EAGLE-3 data (micro-ITT population)

Qualifying uropathogen genotype/clone*^a^*	Treatment group		Total N (%)*^b^* *N* = 1,421
	Gepotidacin N (%)*^b^* *N* = 732	Nitrofurantoin N (%)*^b^* *N* = 689	
Total number of qualifying uropathogens recovered	764	722	1,486
*Escherichia coli*	598 (78)	561 (78)	1,159 (78)
*β*-lactamase gene-positive	102 (17)	87 (16)	189 (16)
Narrow-spectrum *β*-lactamases	36 (6)	30 (5)	66 (6)
EC-5	11 (2)	6 (1)	17 (1)
TEM-1	22 (4)	21 (4)	43 (4)
ESBLs	86 (14)	69 (12)	155 (13)
CTX-M-15	25 (4)	17 (3)	42 (4)
CTX-M-15, OXA-1_OXA-30	23 (4)	17 (3)	40 (3)
CTX-M-27	25 (4)	19 (3)	44 (4)
Extended-spectrum AmpCs	61 (10)	44 (8)	105 (9)
EC-6	52 (9)	32 (6)	84 (7)
EC-6-like	7 (1)	10 (2)	17 (1)
Plasmid AmpCs	5 (<1)	6 (1)	11 (<1)
Uncategorized-spectrum *β*-lactamases	29 (5)	34 (6)	63 (5)
EC	5 (<1)	6 (1)	11 (<1)
EC-11-like	7 (1)	6 (1)	13 (1)
EC-26-like	7 (1)	6 (1)	13 (1)
QRDR mutations	184 (31)	158 (28)	342 (30)
*gyrA* mutations	184 (31)	158 (28)	342 (30)
S83L	17 (3)	12 (2)	29 (3)
S83L, D87N	166 (28)	144 (26)	310 (27)
*parC* mutations	173 (29)	147 (26)	320 (28)
S80I	95 (16)	91 (16)	186 (16)
S80I, E84V	71 (12)	53 (9)	124 (11)
*parE* mutations	50 (8)	48 (9)	98 (8)
L416F	50 (8)	48 (9)	98 (8)
PMQR gene-positive	55 (9)	40 (7)	95 (8)
*aac(6’)-Ib-cr*	27 (5)	20 (4)	47 (4)
*qnrS1*	12 (2)	6 (1)	18 (2)
Epidemiological clones			
ST10	11 (2)	13 (2)	24 (2)
ST1193	25 (4)	28 (5)	53 (5)
ST12	7 (1)	7 (1)	14 (1)
ST127	4 (<1)	10 (2)	14 (1)
ST131	62 (10)	49 (9)	111 (10)
ST141	2 (<1)	8 (1)	10 (<1)
ST69	11 (2)	19 (3)	30 (3)
ST73	18 (3)	18 (3)	36 (3)
ST95	10 (2)	14 (2)	24 (2)
O-type nontypeable:H5	6 (1)	6 (1)	12 (1)
O25b:H4	45 (8)	31 (6)	76 (7)
*fimH*30	41 (7)	31 (6)	72 (6)
*Klebsiella pneumoniae*	56 (7)	58 (8)	114 (8)
*β*-lactamase gene-positive	11 (20)	8 (14)	19 (17)
Narrow-spectrum *β*-lactamases	11 (20)	8 (14)	19 (17)
ESBLs	8 (14)	6 (10)	14 (12)
QRDR mutations	5 (9)	7 (12)	12 (11)
*gyrA* mutations	5 (9)	7 (12)	12 (11)
*parC* mutations	5 (9)	7 (12)	12 (11)
S80I	5 (9)	7 (12)	12 (11)
PMQR gene-positive	13 (23)	12 (21)	25 (22)
*Proteus mirabilis*	34 (4)	33 (5)	67 (5)
QRDR mutations	11 (32)	3 (9)	14 (21)
*gyrA* mutations	11 (32)	3 (9)	14 (21)
*parC* mutations	11 (32)	3 (9)	14 (21)
S84I	11 (32)	1 (3)	12 (18)

^*a*^ Only selected baseline qualifying uropathogens (present at ≥10^5^ CFU/mL) and/or genotypic subcategories with *n* ≥ 10 isolates for the pooled trials and both treatment groups combined are displayed. Only a subset of isolates was selected for genotypic characterization based on predefined phenotypic/MIC criteria (Supplemental Material). Only genotypes with *n* ≥ 5 isolates for the pooled trials and both treatment groups combined are displayed for the MLST and O:H genotypic categories.

^*b*^ Percentage of each qualifying uropathogen was calculated using the total number of baseline qualifying uropathogens at baseline as the denominator. Percentage of each genotypic subcategory was calculated using the number of each respective baseline qualifying uropathogen (present at ≥10^5^ CFU/mL) as the denominator in a post hoc analysis. For MLSTs, O:H serotypes, and *fimH* alleles, percentages do not reflect prevalence among each species as only a selected subset of isolates were tested (based on predefined phenotypic/MIC criteria [Supplemental Material] and additionally, all microbiological failure isolates were characterized by MLST). Uropathogens may have more than one genotype. Bacterial species are only shown for qualifying uropathogens with a sufficient number of genotypes to display; refer to the drug-resistant phenotype co-publication for these additional uropathogens (21).

Among *K. pneumoniae* isolates, 22% were PMQR gene-positive and 11% had QRDR mutations (Table 1; Table S1). All *K. pneumoniae* isolates with QRDR mutations detected harbored both *gyrA* and *parC* mutations; all of the *parC* mutations were S80I. Overall, 17% of *K. pneumoniae* isolates were β-lactamase gene-positive, with ESBLs detected in 12% of isolates.

Among *Proteus mirabilis* isolates, QRDR mutations were the most common genotype (21% of all *P. mirabilis* isolates; Table 1; Table S1). Of the 14 *P. mirabilis* isolates that possessed QRDR mutations, all had double mutations in both the *gyrA* and *parC* genes; the most prevalent mutation was *parC* S84I (12 isolates).

### *In vitro* activity of gepotidacin against genotypic subcategories

In the micro-ITT population, based on recently established FDA breakpoints and interpretive criteria for gepotidacin (22), the vast majority of baseline qualifying Enterobacterales isolates and genotypic subcategories were susceptible (MICs ≤ 16 µg/mL) and none were resistant (MIC ≥ 64 µg/mL) to gepotidacin (Tables 2 and 3).

**TABLE 2:** *In vitro* activity of gepotidacin against selected baseline qualifying uropathogens and genotypic subcategories with *n* ≥ 10 isolates across treatment groups for pooled EAGLE-2 and EAGLE-3 data (micro-ITT population)

Uropathogen genotype/clone*^a^*	No. of isolates	Gepotidacin MIC (µg/mL)		
		Range	MIC_50_	MIC_90_
*Escherichia coli*	1,159	≤0.03 to 32	1	4
*β*-lactamase gene-positive	189	0.12 to 16	2	4
Narrow-spectrum *β*-lactamases	66	0.5 to 16	1	4
EC-5	17	0.5 to 4	1	2
TEM-1	43	0.5 to 16	1	4
ESBLs	155	0.12 to 16	2	4
CTX-M-15	42	0.25 to 16	2	8
CTX-M-15, OXA-1_OXA-30	40	0.5 to 16	2	2
CTX-M-27	44	0.12 to 8	2	4
Extended-spectrum AmpCs	105	0.5 to 16	2	4
EC-6	84	0.5 to 8	2	4
EC-6-like	17	1 to 4	1	2
Plasmid AmpCs	11	0.5 to 16	4	8
Uncategorized-spectrum *β*-lactamases	63	0.25 to 16	2	8
EC	11	0.25 to 8	1	4
EC-11-like	13	0.5 to 8	2	8
EC-26-like	13	0.5 to 8	2	8
QRDR mutations	342	≤0.03 to 16	1	4
*gyrA* mutations	342	≤0.03 to 16	1	4
S83L	29	0.5 to 16	2	4
S83L, D87N	310	≤0.03 to 16	1	4
*parC* mutations	320	≤0.03 to 16	1	4
S80I	186	≤0.03 to 16	1	2
S80I, E84V	124	0.5 to 8	2	4
*parE* mutations	98	≤0.03 to 4	0.5	1
L416F	98	≤0.03 to 4	0.5	1
PMQR gene-positive	95	0.5 to 32	2	8
*aac(6’)-Ib-cr*	47	0.5 to 4	2	4
*qnrS1*	18	1 to 32	8	16
Epidemiological clones				
ST10	24	0.25 to 16	1	4
ST1193	53	≤0.03 to 4	0.5	1
ST12	14	0.5 to 16	1	8
ST127	14	1 to 4	2	2
ST131	111	0.5 to 8	2	4
ST141	10	1 to 2	1	2
ST69	30	0.5 to 8	1	2
ST73	36	0.5 to 4	2	4
ST95	24	1 to 4	1	2
O-type nontypeable:H5	12	0.12 to 2	0.5	2
O25b:H4	76	0.5 to 8	2	4
*fimH*30	72	0.5 to 8	2	4
*Klebsiella pneumoniae*	114	1 to 32	4	16
*β*-lactamase gene-positive	19	2 to 32	4	32
Narrow-spectrum *β*-lactamases	19	2 to 32	4	32
ESBLs	14	2 to 16	4	16
QRDR mutations	12	2 to 16	4	8
*gyrA* mutations	12	2 to 16	4	8
*parC* mutations	12	2 to 16	4	8
S80I	12	2 to 16	4	8
PMQR gene-positive	25	2 to 32	8	32
*Proteus mirabilis*	67	1 to 32	8	16
QRDR mutations	14	1 to 8	4	8
*gyrA* mutations	14	1 to 8	4	8
*parC* mutations	14	1 to 8	4	8
S84I	12	1 to 8	4	8

^*a*^ Only selected baseline qualifying uropathogens (present at ≥10^5^ CFU/mL) and/or genotypic subcategories with *n* ≥ 10 isolates for the pooled trials and both treatment groups combined are displayed. Only genotypes with *n* ≥ 5 isolates for the pooled trials and both treatment groups combined are displayed for the MLST and O:H genotypic categories. Uropathogens may have more than one genotype. Bacterial species are only shown for qualifying uropathogens with a sufficient number of genotypes to display; refer to the drug-resistant phenotype co-publication for these additional uropathogens (21).

**TABLE 3:** Gepotidacin MIC frequency distribution against selected baseline qualifying uropathogens and genotypic subcategories with *n* ≥ 10 isolates across treatment groups for pooled EAGLE-2 and EAGLE-3 data (micro-ITT population)

Qualifying uropathogen genotype/clone*^a^*	No. of isolates^*b*^	No. of isolates/(cumulative %) inhibited with gepotidacin MIC (µg/mL) of*^c^*											MIC_50_	MIC_90_
		≤0.03	0.06	0.12	0.25	0.5	1	2	4	8	16	32		
*Escherichia coli*	1,159	1 (<0.1)	0 (<0.1)	2 (0.03)	11 (1.2)	101 (9.9)	502 (53.2)	424 (89.8)	93 (97.8)	17 (99.9)	7 (>99.9)	1 (100)	1	4
*β*-lactamase gene-positive	189			1 (0.5)	2 (1.6)	19 (11.6)	68 (47.6)	66 (82.5)	20 (93.1)	9 (97.9)	4 (100)		2	4
Narrow-spectrum *β*-lactamases	66					13 (19.7)	25 (57.6)	17 (83.3)	8 (95.5)	2 (98.5)	1 (100)		1	4
EC-5	17					6 (35.3)	6 (70.6)	4 (94.1)	1 (100)				1	2
TEM-1	43					5 (11.6)	18 (53.5)	11 (79.1)	7 (95.3)	1 (97.7)	1 (100)		1	4
ESBLs	155			1 (0.6)	2 (1.9)	15 (11.6)	53 (45.8)	56 (81.9)	16 (92.3)	8 (97.4)	4 (100)		2	4
CTX-M-15	42				1 (2.4)	3 (9.5)	12 (38.1)	10 (61.9)	7 (78.6)	6 (92.9)	3 (100)		2	8
CTX-M-15, OXA-1_OXA-30	40					1 (2.5)	16 (42.5)	19 (90.0)	2 (95.0)	1 (97.5)	1 (100)		2	2
CTX-M-27	44			1 (2.3)	1 (4.5)	9 (25.0)	10 (47.7)	16 (84.1)	6 (97.7)	1 (100)			2	4
Extended-spectrum AmpCs	105					7 (6.7)	39 (43.8)	43 (84.8)	13 (97.1)	2 (99.0)	1 (100)		2	4
EC-6	84					7 (8.3)	27 (40.5)	36 (83.3)	12 (97.6)	2 (100)			2	4
EC-6-like	17						10 (58.8)	6 (94.1)	1 (100)				1	2
Plasmid AmpCs	11					2 (18.2)	1 (27.3)	1 (36.4)	4 (72.7)	2 (90.9)	1 (100)		4	8
Uncategorized-spectrum *β-*lactamases	63				1 (1.6)	4 (7.9)	23 (44.4)	19 (74.6)	6 (84.1)	7 (95.2)	3 (100)		2	8
EC	11				1 (9.1)	0 (9.1)	5 (54.5)	1 (63.6)	3 (90.9)	1 (100)			1	4
EC-11-like	13					1 (7.7)	4 (38.5)	4 (69.2)	2 (84.6)	2 (100)			2	8
EC-26-like	13					1 (7.7)	4 (38.5)	6 (84.6)	0 (84.6)	2 (100)			2	8
QRDR mutations	342	1 (0.3)	0 (0.3)	2 (0.9)	8 (3.2)	61 (21.1)	122 (56.7)	100 (86.0)	40 (97.7)	6 (99.4)	2 (100)		1	4
*gyrA* mutations	342	1 (0.3)	0 (0.3)	2 (0.9)	8 (3.2)	61 (21.1)	122 (56.7)	100 (86.0)	40 (97.7)	6 (99.4)	2 (100)		1	4
S83L	29					3 (10.3)	10 (44.8)	8 (72.4)	7 (96.6)	0 (96.6)	1 (100)		2	4
S83L, D87N	310	1 (0.3)	0 (0.3)	1 (0.6)	8 (3.2)	58 (21.9)	111 (57.7)	92 (87.4)	32 (97.7)	6 (99.7)	1 (100)		1	4
*parC* mutations	320	1 (0.3)	0 (0.3)	2 (0.9)	8 (3.4)	59 (21.9)	115 (57.8)	95 (87.5)	33 (97.8)	6 (99.7)	1 (100)		1	4
S80I	186	1 (0.5)	0 (0.5)	2 (1.6)	8 (5.9)	54 (34.9)	74 (74.7)	37 (94.6)	6 (97.8)	3 (99.5)	1 (100)		1	2
S80I, E84V	124					4 (3.2)	36 (32.3)	55 (76.6)	26 (97.6)	3 (100)			2	4
*parE* mutations	98	1 (1.0)	0 (1.0)	1 (2.0)	7 (9.2)	44 (54.1)	37 (91.8)	6 (98.0)	2 (100)				0.5	1
L416F	98	1 (1.0)	0 (1.0)	1 (2.0)	7 (9.2)	44 (54.1)	37 (91.8)	6 (98.0)	2 (100)				0.5	1
PMQR gene-positive	95					1 (1.1)	22 (24.2)	34 (60.0)	19 (80.0)	12 (92.6)	6 (98.9)	1 (100)	2	8
*aac(6’)-Ib-cr*	47					1 (2.1)	16 (36.2)	25 (89.4)	5 (100)				2	4
*qnrS1*	18						1 (5.6)	1 (11.1)	4 (33.3)	7 (72.2)	4 (94.4)	1 (100)	8	16
Epidemiological clones														
ST10	24				2 (8.3)	0 (8.3)	12 (58.3)	4 (75.0)	4 (91.7)	1 (95.8)	1 (100)		1	4
ST1193	53	1 (1.9)	0 (1.9)	1 (3.8)	3 (9.4)	22 (50.9)	22 (92.5)	3 (98.1)	1 (100)				0.5	1
ST12	14					2 (14.3)	7 (64.3)	3 (85.7)	0 (85.7)	1 (92.9)	1 (100)		1	8
ST127	14						6 (42.9)	7 (92.9)	1 (100)				2	2
ST131	111					7 (6.3)	42 (44.1)	43 (82.9)	17 (98.2)	2 (100)			2	4
ST141	10						6 (60.0)	4 (100)					1	2
ST69	30					1 (3.3)	18 (63.3)	9 (93.3)	1 (96.7)	1 (100)			1	2
ST73	36					2 (5.6)	12 (38.9)	18 (88.9)	4 (100)				2	4
ST95	24						15 (62.5)	7 (91.7)	2 (100)				1	2
O-type nontypeable:H5	12			1 (8.3)	1 (16.7)	4 (50.0)	4 (83.3)	2 (100)					0.5	2
O25b:H4	76					5 (6.6)	23 (36.8)	35 (82.9)	11 (97.4)	2 (100)			2	4
*fimH*30	72					5 (6.9)	23 (38.9)	31 (81.9)	11 (97.2)	2 (100)			2	4
*Klebsiella pneumoniae*	114						1 (0.9)	13 (12.3)	53 (58.8)	34 (88.6)	9 (96.5)	4 (100)	4	16
*β*-lactamase gene-positive	19							2 (10.5)	8 (52.6)	3 (68.4)	4 (89.5)	2 (100)	4	32
Narrow-spectrum *β*-lactamases	19							2 (10.5)	8 (52.6)	3 (68.4)	4 (89.5)	2 (100)	4	32
ESBLs	14							2 (14.3)	6 (57.1)	3 (78.6)	3 (100)		4	16
QRDR mutations	12							3 (25.0)	7 (83.3)	1 (91.7)	1 (100)		4	8
*gyrA* mutations	12							3 (25.0)	7 (83.3)	1 (91.7)	1 (100)		4	8
*parC* mutations	12							3 (25.0)	7 (83.3)	1 (91.7)	1 (100)		4	8
S80I	12							3 (25.0)	7 (83.3)	1 (91.7)	1 (100)		4	8
PMQR gene-positive	25							3 (12.0)	8 (44.0)	5 (64.0)	5 (84.0)	4 (100)	8	32
*Proteus mirabilis*	67						3 (4.5)	4 (10.4)	15 (32.8)	31 (79.1)	11 (95.5)	3 (100)	8	16
QRDR mutations	14						3 (21.4)	3 (42.9)	5 (78.6)	3 (100)			4	8
*gyrA* mutations	14						3 (21.4)	3 (42.9)	5 (78.6)	3 (100)			4	8
*parC* mutations	14						3 (21.4)	3 (42.9)	5 (78.6)	3 (100)			4	8
S84I	12						2 (16.7)	2 (33.3)	5 (75.0)	3 (100)			4	8

^*a*^ Only selected baseline qualifying uropathogens (present at ≥10^5^ CFU/mL) and/or genotypic subcategories with *n* ≥ 10 isolates for the pooled trials and both treatment groups combined are displayed. Uropathogens may have more than one genotype.

^*b*^ Number of isolates with nonmissing MIC values.

^*c*^ The percentage of isolates shown at each gepotidacin MIC concentration was calculated as a cumulative value in a post hoc analysis. Gepotidacin MIC values ranged from ≤0.03 to 32 µg/mL; MIC columns for 64 µg/mL and >64 µg/mL are not shown as there were no data to present.

In the micro-ITT population, the gepotidacin MIC_90_ for all *E. coli* isolates was 4 µg/mL (>99.9% of isolates were susceptible to gepotidacin) (Tables 2 and 3) (22). MIC_90_ values for gepotidacin ranged from 1 to 16 µg/mL (±2-dilutions of the overall MIC_90_) against genotypic subsets of *E. coli* isolates (*n* ≥ 10), with the majority (94.4%) of isolates susceptible to gepotidacin. Gepotidacin MIC_90_ values were 4 µg/mL or lower for the majority of *E. coli* genotypic categories, with some exceptions, including CTX-M-15 and PMQR gene-positive genotypes with gepotidacin MIC_90_ values of 8 µg/mL (1-dilution higher compared with *E. coli* isolates overall, 100% and 98.9% of isolates, respectively, were susceptible to gepotidacin) and the PMQR *qnrS1* genotype, which had a gepotidacin MIC_90_ of 16 µg/mL (2-dilutions higher in comparison to *E. coli* isolates overall, 94.4% were susceptible to gepotidacin). This collection of isolates may not reflect the complete picture of uUTI epidemiology, as only a subset was selected for testing based on predefined phenotypic/MIC criteria and for all cases of microbiological failure (Supplemental material).

The gepotidacin MIC_90_ for all *K. pneumoniae* isolates was 16 µg/mL, with 96.5% of isolates susceptible to gepotidacin (Tables 2 and 3) (22). MIC_90_ values for gepotidacin ranged from 8 to 32 µg/mL (±1-dilution of the overall MIC_90_), and the percent of isolates susceptible to gepotidacin ranged from 84.0% to 100% against genotypic subsets of *K. pneumoniae* isolates (*n* ≥ 10). Gepotidacin MIC_90_ values were 8 to 16 µg/mL against the majority of *K. pneumoniae* genotypic subcategories, with the exception of narrow-spectrum β-lactamases and PMQR gene-positive isolates (MIC_90_ = 32 µg/mL), with 89.5% and 84.0% of isolates, respectively, susceptible to gepotidacin.

The gepotidacin MIC_90_ for all *P. mirabilis* isolates was 16 µg/mL, with 95.5% of isolates susceptible to gepotidacin (Tables 2 and 3) (22). MIC_90_ values for gepotidacin were 8 µg/mL for isolates with QRDR mutations (*n* ≥ 10). All *P. mirabilis* isolates with QRDR mutations were considered susceptible to gepotidacin.

No baseline qualifying uropathogens with *gyrA* D82N or *parC* D79N mutations (QRDR mutations associated with gepotidacin binding) were identified (15, 16).

### Gepotidacin efficacy against genotypic subcategories

**Fig 1:**
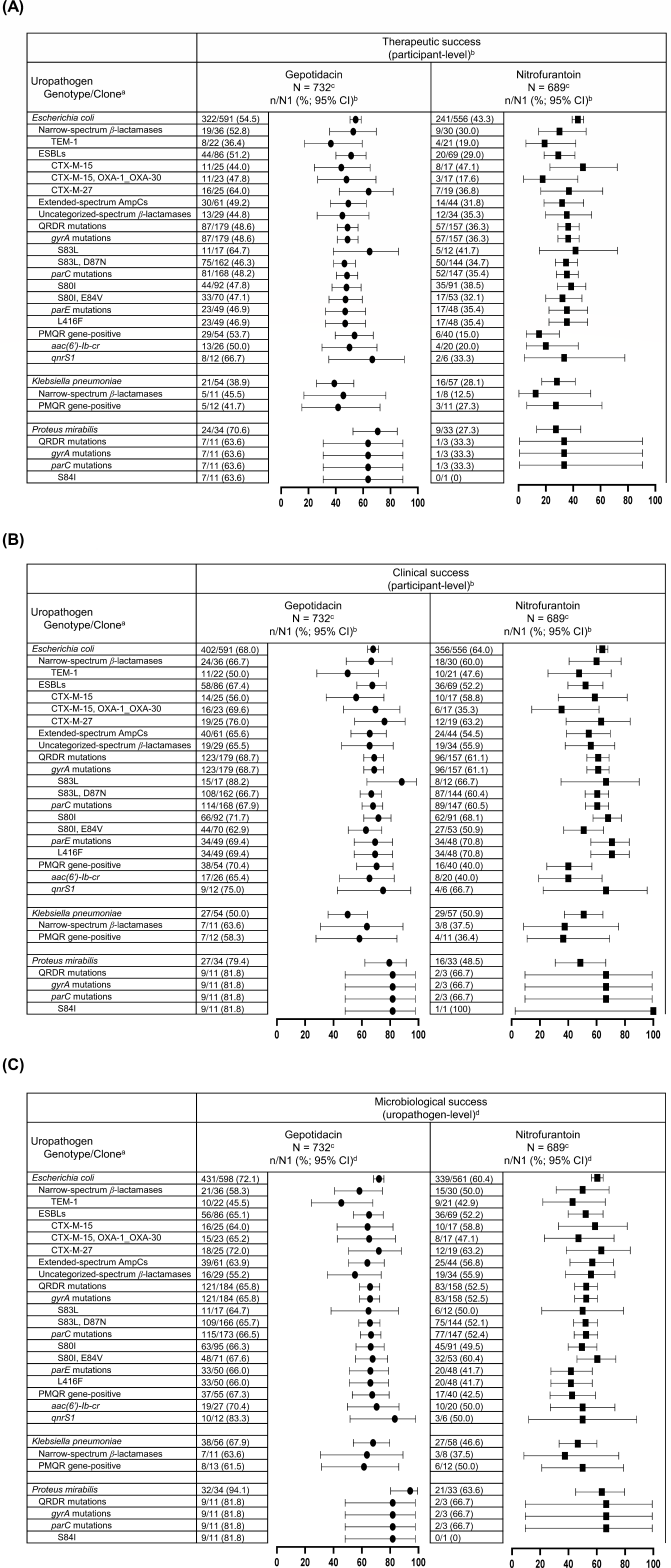
Therapeutic (**A**), clinical (**B**), and microbiological (**C**) success at TOC by selected baseline qualifying uropathogens and genotypic subcategories with *n* ≥ 10 participants for at least one treatment group for pooled EAGLE-2 and EAGLE-3 data (micro-ITT population). Footnotes: *^a^*Only select baseline qualifying uropathogens (present at ≥10^5^ CFU/mL) and genotypic subcategories with *n* ≥ 10 isolates for the pooled trials in at least one treatment group are displayed. Uropathogens may have more than one genotype. Of note, epidemiological clone results for *E. coli* are not included as the subset of isolates for molecular characterization included all microbiological failure isolates; therefore, the efficacy data are biased toward failure. *^b^*For clinical and therapeutic response, a participant was counted once under a uropathogen category if multiple qualifying uropathogens within that category were isolated at baseline for the participant. Participants for whom all uropathogens were not eradicated and all symptoms were not resolved were considered therapeutic failures for all uropathogens. For therapeutic and clinical success: n/N1 = (n) the number of participants within the category with a response of success/(N1) the total number of participants within the category, and is the denominator for corresponding percentages. *^c^*The N in the header represents the total number of participants in the treatment arm. *^d^*For microbiological response, a participant was counted more than once under a uropathogen category if multiple qualifying uropathogens within that category were isolated at baseline for the participant. For microbiological success: n/N1 = (n) the number of isolates that are a microbiological success/(N1) the total number of isolates in the category, and is the denominator for the corresponding percentages.

In the pooled micro-ITT population, gepotidacin therapeutic, clinical, and microbiological success rates at TOC were generally similar across genotypic subcategories of each species, although sample sizes were small for some *E. coli* and all *K. pneumoniae* and *P. mirabilis* genotypic subcategories (Fig. 1). No resistance genotypes were disproportionately associated with therapeutic failure. In this population, which included isolates resistant to nitrofurantoin, the majority of success rates were numerically higher in the gepotidacin group compared with the nitrofurantoin group. Similar efficacy trends were observed in the microbiologically evaluable (ME) population at TOC (ME-TOC population; Table S2) and in the ME population at Follow-up (ME-FU population; Table S3).

## DISCUSSION

Pooled microbiology uUTI uropathogen data and genotypic analysis results from the EAGLE-2 and EAGLE-3 trials provide valuable epidemiological and efficacy data to the scientific community. These trials were conducted in 15 countries in North America, Europe, and Asia Pacific, and microbiological data were based on the analysis of urine culture results from specimens collected at baseline (pretreatment) from all enrolled female participants (15). While the majority of uropathogens were recovered from participants in the US and Bulgaria (15), this large set of genotypic data affords insight into causative uUTI pathogens and their genotypic characteristics.

The results and discussion presented are focused primarily on *E. coli* genotypes due to their high prevalence in the pooled data set. *K. pneumoniae* and *P. mirabilis* had sample sizes of 8% and 5% of overall isolates, respectively, but the number of genotypic subcategories was small (Table 2). Although data were pooled across 2 uUTI phase 3 trials, the percentage of several other bacterial species and associated genotypes was also small for detailed assessments.

The most prominent uropathogen, *E. coli*, had a robust prevalence of genotypic subcategories for interpretation in the pooled data, including multiple QRDR mutations, numerous β-lactamase resistance genes (primarily ESBLs), the pathogenic circulating multidrug-resistant ST131 clone, and the *fimH*30 subclone. Molecular characterization was performed on a subset of isolates per predefined phenotypic/MIC criteria, and additionally, for MLST, included all isolates associated with microbiological failure; therefore, the incidence of genotypes reported herein does not represent the overall prevalence among uropathogens recovered from uUTIs. Nonetheless, the diverse genotypic mechanisms of β-lactam and fluoroquinolone resistance among *E. coli* observed in the trials confirm the need for antibacterials with novel mechanisms of action for the treatment of uUTI caused by this priority uropathogen (23, 24).

The vast majority of genotypic subcategories of Enterobacterales in the study were susceptible (MICs ≤ 16 µg/mL) to gepotidacin based on the FDA’s recently published breakpoints and interpretive criteria (22). Across a broad range of *E. coli* genotypes, gepotidacin was active with MIC_90_ values ranging from 1 to 8 µg/mL, including isolates with a PMQR *qnrS1* genotype (MIC_90_ = 16 µg/mL), and with a considerable percentage of isolates susceptible to gepotidacin (94.4% to 100%; Tables 2 and 3). These findings generally align with previously reported gepotidacin *in vitro* activity against *E. coli* isolates (17, 25). Against the pathogenic circulating multidrug-resistant *E. coli* ST131 clone, including *fimH*30 and O25b:H4 subclones, notable gepotidacin *in vitro* activity was observed (MIC_90_ = 4 µg/mL; 100% of isolates susceptible to gepotidacin). The *in vitro* activity of gepotidacin was associated with appreciable rates of microbiological response at TOC in the pooled micro-ITT (72.1%) and ME-TOC (82.7%) populations for *E. coli* isolates overall. Across uropathogen genotypes, gepotidacin MIC_90_ values were generally similar (i.e., lower, equal to, or 1-dilution higher) when compared with the MIC_90_ value for the overall species, except for *E. coli* isolates with PMQR *qnrS1*, for which the MIC_90_ values were 2-dilution higher.

While data suggest that *qnrS1* is associated with higher gepotidacin MICs against *E. coli*, given the wide range of gepotidacin MICs for *qnr* genotypes seen, higher gepotidacin MICs were not able to be attributed to a single mechanism. None of the genotypes described resulted in significant pre-existing reduced susceptibility to gepotidacin, as evidenced by the *in vitro* activity data presented (Tables 2 and 3), where 94.4% of *E. coli* isolates with *qnr* genotypes were susceptible to gepotidacin (MIC ≤ 16 µg/mL; 22). In addition, gepotidacin largely maintained efficacy against isolates carrying these genotypes.

Gepotidacin and fluoroquinolones both target the same enzymes (DNA gyrase and topoisomerase IV); however, gepotidacin inhibits bacterial DNA replication by a distinct binding site, a novel mechanism of action, and for most pathogens, provides well-balanced inhibition of both enzymes (13–16). These attributes support gepotidacin’s consistent *in vitro* activity (Table 3) against drug-resistant genotypes, including isolates with QRDR mutations, and a lack of target-specific cross-resistance with fluoroquinolones and other antibacterials.

Due to its well-balanced inhibition, gepotidacin has a low potential for resistance development as gepotidacin target-specific mutations in both DNA gyrase and topoisomerase IV enzymes are needed to significantly affect gepotidacin susceptibility (15, 16). In *E. coli* and *K. pneumoniae,* the two residues that have been associated with gepotidacin binding are *gyrA* D82N and ParC D79N (14–16). The following amino acids may also be important for gepotidacin activity as shown through studies with isogenic mutants in *E. coli* and *K. pneumoniae: gyrA* P35, V44, and A175, and *gyrB* D426 and P445 (20). Mutations in these residues were not observed in the subset of isolates molecularly characterized in the EAGLE-2 or EAGLE-3 uUTI trials.

Of note, additional aspects of these data are still to be explored and reported. FDA breakpoints and interpretive criteria for gepotidacin were recently published for Enterobacterales, *S. saprophyticus*, and *E. faecalis* uropathogens (22). However, evaluation of the *in vitro* activity and clinical efficacy data for gepotidacin may be of interest when additional officially recognized breakpoints become available. Further analyses relevant to the establishment of clinical breakpoints (e.g., outcome-by-MIC analyses, clinical exposure/response, and probability of target attainment) will be considered for future publication.

These findings show gepotidacin’s efficacy and *in vitro* activity against a broad range of uUTI uropathogen genotypes. The microbiological data presented represents a robust and contemporary (2019 to 2022) data set that provides insight into the current genotypes of uropathogens. This underscores the ongoing need for new oral treatment options for uUTIs as antibiotic resistance mechanisms continue to present challenges to effective treatment.

## MATERIALS AND METHODS

### Study overview

EAGLE-2 and EAGLE-3 were phase 3 uUTI clinical trials comparing the efficacy and safety of gepotidacin versus nitrofurantoin. The study design and other key aspects of the study population, randomization, study treatments, and ethical standards have been detailed elsewhere (19, 26). Efficacy and safety assessments were performed at the Baseline (day 1; pretreatment), On-therapy (days 2 to 4), TOC (days 10 to 13), and FU (day 28 ± 3) visits.

### Microbiological assessments

Urine sample collection, quantitative urine culture, uropathogen identification, and phenotypic antibacterial susceptibility testing details are reported in the co-publication (21). Briefly, microbiological analyses were performed at a central laboratory (PPD Laboratory Services Central Lab, Highland Heights, KY, USA) using standard procedures and Clinical and Laboratory Standards Institute guidelines for susceptibility testing (27, 28). Gepotidacin susceptibility interpretations were not available at the time of the study but are briefly described in the text according to recently published FDA breakpoints (22).

Element Iowa City (JMI Laboratories; North Liberty, IA, USA) performed molecular genotyping and characterization for a subset of the isolates recovered at baseline per a predefined phenotypic algorithm based on MIC results for specific antibacterial agents against certain bacterial species and for the same uropathogen species recovered at multiple study time points (Supplemental material). Whole genome sequencing (WGS) was performed to screen for antibiotic resistance markers, including β-lactamase genes (i.e., ESBLs, other β-lactamases, plasmid AmpC, and carbapenemases), mutations in the QRDR regions (i.e., *gyrA* and *gyrB* that encode DNA gyrase and *parC* and *parE* that encode topoisomerase IV), and the presence of PMQR genes. A clonal assessment of selected uropathogens (i.e., Enterobacterales with ceftriaxone or meropenem MIC results of ≥2 µg/mL and isolates of the same species recovered at multiple study visits) was determined using MLST, or pulsed-field gel electrophoresis if an MLST scheme was not available for a species. Selected *E. coli* isolates with ceftriaxone or meropenem MIC results of ≥2 µg/mL were evaluated by O:H serotyping (29), and for those isolates determined to be ST131 by MLST, the *fimH* gene allele was also investigated.

For WGS, the total genomic DNA of selected isolates was extracted using KingFisher Flex magnetic particle processors (ThermoFisher, Cleveland, OH, USA) and used as input material for DNA library construction using the Nextera XT Library Preparation Kit (Illumina, San Diego, California, USA), followed by sequencing via the MiSeq System (Illumina, San Diego, California, USA); all performed per manufacturer’s instructions. DNA was assembled *de novo* using SPAdes version 3.9.0 and subjected to proprietary software (Element Iowa City) for screening of resistance genes. Genomes were paired against curated databases containing resistance genes. QRDR genes were extracted and paired with sequences from a susceptible wild-type and reference isolate to screen for mutations. Reference isolates for comparison comprised *E. coli* ATCC25922, *K. pneumoniae* ATCC13883, *P. mirabilis* ATCC29906, *Enterobacter cloacae* ATCC13047, *C. freundii* ATCC8090, *Morganella morganii* ATCC8076H, and *Serratia marcescens* ATCC13880.

For the expression of AmpC and AcrAB, as applicable, total mRNA was extracted and purified using the RNeasy Mini Kit and QIAcube workstation (both from QIAGEN, Germantown, MD, USA) according to the manufacturer’s instructions. Residual DNA was eliminated by treatment with RNAse-free DNase (Promega, Madison, WI, USA). Sample quality and quantification were assessed using the Agilent RNA 6000 Nano kit and Agilent 2100 Bioanalyzer (Agilent, Santa Clara, CA, USA) per manufacturer’s instructions. Transcription levels of *ampC* and *acrAB* were determined by PCR using the Step-One Plus Real-Time PCR System (ThermoFisher Scientific, Waltham, MA, USA) and standard protocols.

For molecular typing, assembled genomes of Enterobacterales strains that met the testing criteria for β-lactamases were targeted for extraction per predefined sets of seven housekeeping gene fragments (about 500 bp). Each fragment was compared with known allele variants for each locus (housekeeping gene) on the MLST website (http://www.mlst.net). Unassigned loci and MLST profiles were submitted to the MLST website. Pulsed-field gel electrophoresis was performed using standard methods (30).

### Statistical analysis

The micro-ITT population definition, as well as definitions of baseline qualifying uropathogens, and therapeutic, clinical, and microbiological outcome and response, have been previously described (19). The ME-TOC and ME-FU populations are defined in Tables S2 and S3, respectively.

Statistical analyses were performed using SAS. For the incidence of each genotypic subcategory for each uropathogen, percentages were calculated using the number of isolates in each genotypic category as the numerator and the number of isolates for each respective baseline qualifying uropathogen species (present at ≥10^5^ CFU/mL) as the denominator in a post hoc analysis. These percentages serve as a representative sample of distribution and do not reflect overall prevalence for the genotypes, and as an example, only a selected subset of isolates was tested for MLST (based on predefined phenotypic/MIC criteria [Supplemental material] plus all microbiological failure isolates).

Therapeutic, clinical, and microbiological success rates, along with the 95% Exact Clopper-Pearson confidence intervals, were determined as previously described (21).

## Data Availability

Anonymized individual participant data and study documents can be requested for further research from https://www.gsk-studyregister.com/en/.

## SUPPLEMENTAL MATERIAL

Supplemental material aac.01639-24-s0001.docx Tables S1 to S3.

ASM does not own the copyrights to Supplemental Material that may be linked to, or accessed through, an article. The authors have granted ASM a non-exclusive, world-wide license to publish the Supplemental Material files. Please contact the corresponding author directly for reuse.
