# Supplementary material for: Efficacy and in vitro activity of gepotidacin against bacterial uropathogens, including subsets with molecularly characterized resistance mechanisms and genotypes/epidemiological clones, in females with uncomplicated urinary tract infections: results from two global, pivotal, phase 3 trials (EAGLE-2 and EAGLE-3)
Source: Antimicrob Agents Chemother. 2025 Sep 9;69(10):e01639-24. doi: 10.1128/aac.01639-24 (PMC12486806; doi:10.1128/aac.01639-24)
Supplement: Supplemental material — Tables S1 to S3. [file aac.01639-24-s0001.docx]

**Supplemental Material**

**Efficacy and *In Vitro* Activity of Gepotidacin Against Bacterial Uropathogens, Including Subsets with Molecularly Characterized Resistance Mechanisms and Genotypes/Epidemiological Clones, in Females with Uncomplicated Urinary Tract Infections: Results from 2 Global, Pivotal, Phase 3 Trials (EAGLE-2 and EAGLE-3)**

Nicole E. Scangarella-Oman, Deborah L. Butler, John Breton, Derrek Brown, Cara Kasapidis, Amanda J. Sheets

**Supplemental Material Contents:**

**Table S1** Incidence of baseline qualifying uropathogens recovered and genotypes/epidemiological clones for pooled EAGLE-2 and EAGLE-3 data (micro-ITT population)

**Table S2** Therapeutic, clinical, and microbiological success at TOC by selected baseline qualifying uropathogens and genotypic subcategories with an *n* ≥10 isolates for at least 1 treatment group for pooled EAGLE-2 and EAGLE-3 data (ME-TOC population)

**Table S3** Therapeutic, clinical, and microbiological success at follow-up by selected baseline qualifying uropathogens and genotypic subcategories with an *n* ≥10 isolates for at least 1 treatment group for pooled EAGLE-2 and EAGLE-3 data (ME-FU population)

**Predefined criteria for molecular characterization for a subset of uropathogen isolates**

**Table S1** Incidence of baseline qualifying uropathogens recovered and genotypes/epidemiological clones for pooled EAGLE-2 and EAGLE-3 data (micro-ITT population)

| **Qualifying uropathogen**  **Genotype/Clone***^b^* | **Treatment group** | | | **Total n (%)*^a^***  **N=1,421** |
| --- | --- | --- | --- | --- |
|  | **Gepotidacin n (%)*^a^***  **N=732** | **Nitrofurantoin n (%)*^a^***  **N=689** | |  |
| Total number of qualifying uropathogens recovered | 764 | | 722 | 1,486 |
|  |  | |  |  |
| *Escherichia coli* | 598 (78) | | 561 (78) | 1,159 (78) |
| *ꞵ*-lactamase gene-positive | 102 (17) | | 87 (16) | 189 (16) |
| Narrow-spectrum *ꞵ*-lactamases | 36 (6) | | 30 (5) | 66 (6) |
| EC-5 | 11 (2) | | 6 (1) | 17 (1) |
| EC-5, TEM-1 | 1 (<1) | | 1 (<1) | 2 (<1) |
| TEM-1 | 22 (4) | | 21 (4) | 43 (4) |
| TEM-190 | 0 | | 1 (<1) | 1 (<1) |
| TEM-210 | 1 (<1) | | 0 | 1 (<1) |
| TEM-35 | 0 | | 1 (<1) | 1 (<1) |
| TEM-40 | 1 (<1) | | 0 (0) | 1 (<1) |
| ESBLs | 86 (14) | | 69 (12) | 155 (13) |
| CTX-M-1 | 2 (<1) | | 0 (0) | 2 (<1) |
| CTX-M-14 | 3 (<1) | | 4 (<1) | 7 (<1) |
| CTX-M-14, OXA-1_OXA-30 | 0 | | 1 (<1) | 1 (<1) |
| CTX-M-15 | 25 (4) | | 17 (3) | 42 (4) |
| CTX-M-15, CTX-M-27, OXA-1_OXA-30 | 0 | | 1 (<1) | 1 (<1) |
| CTX-M-15, OXA-1_OXA-30 | 23 (4) | | 17 (3) | 40 (3) |
| CTX-M-15, OXA-1_OXA-30-like | 1 (<1) | | 0 | 1 (<1) |
| CTX-M-27 | 25 (4) | | 19 (3) | 44 (4) |
| CTX-M-3 | 0 | | 3 (<1) | 3 (<1) |
| CTX-M-55 | 2 (<1) | | 3 (<1) | 5 (<1) |
| CTX-M-65 | 0 | | 1 (<1) | 1 (<1) |
| OXA-1_OXA-30 | 1 (<1) | | 1 (<1) | 2 (<1) |
| SHV-12 | 4 (<1) | | 2 (<1) | 6 (<1) |
| Extended-spectrum AmpCs | 61 (10) | | 44 (8) | 105 (9) |
| EC-18-like | 1 (<1) | | 0 | 1 (<1) |
| EC-19-like | 1 (<1) | | 2 (<1) | 3 (<1) |
| EC-6 | 52 (9) | | 32 (6) | 84 (7) |
| EC-6-like | 7 (1) | | 10 (2) | 17 (1) |
| Plasmid AmpCs | 5 (<1) | | 6 (1) | 11 (<1) |
| CMY-2 | 2 (<1) | | 0 | 2 (<1) |
| CMY-32 | 0 | | 1 (<1) | 1 (<1) |
| CMY-4 | 0 | | 1 (<1) | 1 (<1) |
| CMY-42 | 0 | | 1 (<1) | 1 (<1) |
| DHA-1 | 2 (<1) | | 3 (<1) | 5 (<1) |
| DHA-1-like | 1 (<1) | | 0 | 1 (<1) |
| Carbapenemase | 1 (<1) | | 0 | 1 (<1) |
| OXA-181 | 1 (<1) | | 0 | 1 (<1) |
| Uncategorized-spectrum *ꞵ*-lactamases | 29 (5) | | 34 (6) | 63 (5) |
| EC | 5 (<1) | | 6 (1) | 11 (<1) |
| EC-11-like | 7 (1) | | 6 (1) | 13 (1) |
| EC-12-like | 3 (<1) | | 4 (<1) | 7 (<1) |
| EC-26 | 1 (<1) | | 2 (<1) | 3 (<1) |
| EC-26-like | 7 (1) | | 6 (1) | 13 (1) |
| EC-31 | 1 (<1) | | 4 (<1) | 5 (<1) |
| EC-8-like | 0 | | 2 (<1) | 2 (<1) |
| EC-9-like | 3 (<1) | | 4 (<1) | 7 (<1) |
| EC-like | 2 (<1) | | 0 | 2 (<1) |
| QRDR mutations | 184 (31) | | 158 (28) | 342 (30) |
| *gyrA* mutations | 184 (31) | | 158 (28) | 342 (30) |
| S83A | 0 | | 1 (<1) | 1 (<1) |
| S83L | 17 (3) | | 12 (2) | 29 (3) |
| S83L, D87N | 166 (28) | | 144 (26) | 310 (27) |
| S83L, D87Y | 1 (<1) | | 1 (<1) | 2 (<1) |
| *parC* mutations | 173 (29) | | 147 (26) | 320 (28) |
| E84G | 2 (<1) | | 0 | 2 (<1) |
| E84K | 1 (<1) | | 1 (<1) | 2 (<1) |
| S80I | 95 (16) | | 91 (16) | 186 (16) |
| S80I, A90V | 1 (<1) | | 0 | 1 (<1) |
| S80I, E84G | 2 (<1) | | 0 | 2 (<1) |
| S80I, E84K | 1 (<1) | | 0 | 1 (<1) |
| S80I, E84V | 71 (12) | | 53 (9) | 124 (11) |
| S80R | 0 | | 2 (<1) | 2 (<1) |
| *parE* mutations | 50 (8) | | 48 (9) | 98 (8) |
| L416F | 50 (8) | | 48 (9) | 98 (8) |
| PMQR-gene-positive | 55 (9) | | 40 (7) | 95 (8) |
| *aac(6’)-Ib-cr* | 27 (5) | | 20 (4) | 47 (4) |
| *aac(6’)-Ib-cr, oqxA2, oqxB2* | 0 | | 1 (<1) | 1 (<1) |
| *aac(6’)-Ib-cr, qnrB19* | 2 (<1) | | 0 | 2 (<1) |
| *aac(6’)-Ib-cr, qnrB4* | 0 | | 1 (<1) | 1 (<1) |
| *aac(6’)-Ib-cr, qnr-B4, qnrS1* | 0 | | 1 (<1) | 1 (<1) |
| *aac(6’)-Ib-cr, qnrS1* | 1 (<1) | | 0 | 1 (<1) |
| *oqxA, oqxB* | 2 (<1) | | 0 | 2 (<1) |
| *qepA1* | 0 | | 1 (<1) | 1 (<1) |
| *qepA1*-like | 1 (<1) | | 0 | 1 (<1) |
| *qepA4* | 1 (<1) | | 0 | 1 (<1) |
| *qnrA1* | 0 | | 1 (<1) | 1 (<1) |
| *qnrB19* | 5 (<1) | | 7 (1) | 12 (1) |
| *qnrB19, qnrS1* | 1 (<1) | | 0 | 1 (<1) |
| *qnrB4* | 2 (<1) | | 0 | 2 (<1) |
| *qnrB4, qnrS1* | 1 (<1) | | 1 (<1) | 2 (<1) |
| *qnrS1* | 12 (2) | | 6 (1) | 18 (2) |
| *qnrS2* | 0 | | 1 (<1) | 1 (<1) |
| Epidemiological clones |  | |  |  |
| ST10 | 11 (2) | | 13 (2) | 24 (2) |
| ST1193 | 25 (4) | | 28 (5) | 53 (5) |
| ST12 | 7 (1) | | 7 (1) | 14 (1) |
| ST127 | 4 (<1) | | 10 (2) | 14 (1) |
| ST131 | 62 (10) | | 49 (9) | 111 (10) |
| ST141 | 2 (<1) | | 8 (1) | 10 (<1) |
| ST38 | 4 (<1) | | 5 (<1) | 9 (<1) |
| ST404 | 5 (<1) | | 4 (<1) | 9 (<1) |
| ST405 | 3 (<1) | | 4 (<1) | 7 (<1) |
| ST420 | 4 (<1) | | 5 (<1) | 9 (<1) |
| ST58 | 3 (<1) | | 3 (<1) | 6 (<1) |
| ST69 | 11 (2) | | 19 (3) | 30 (3) |
| ST73 | 18 (3) | | 18 (3) | 36 (3) |
| ST95 | 10 (2) | | 14 (2) | 24 (2) |
| O-type nontypeable:H5 | 6 (1) | | 6 (1) | 12 (1) |
| O16:H5 | 5 (<1) | | 0 | 5 (<1) |
| O25b:H4 | 45 (8) | | 31 (6) | 76 (7) |
| O2:H6 | 0 | | 5 (<1) | 5 (<1) |
| *fimH*27 | 0 | | 1 (<1) | 1 (<1) |
| *fimH*30 | 41 (7) | | 31 (6) | 72 (6) |
| *fimH*41 | 5 (<1) | | 0 | 5 (<1) |
| *fimH*99 | 1 (<1) | | 0 | 1 (<1) |
|  |  | |  |  |
| *Klebsiella pneumoniae* | 56 (7) | | 58 (8) | 114 (8) |
| *ꞵ*-lactamase gene-positive | 11 (20) | | 8 (14) | 19 (17) |
| Narrow-spectrum *ꞵ*-lactamases | 11 (20) | | 8 (14) | 19 (17) |
| SHV-1 | 2 (4) | | 1 (2) | 3 (3) |
| SHV-11 | 2 (4) | | 0 | 2 (2) |
| SHV-11, TEM-1 | 2 (4) | | 1 (2) | 3 (3) |
| SHV-28 | 1 (2) | | 1 (2) | 2 (2) |
| SHV-28, TEM-1 | 2 (4) | | 3 (5) | 5 (4) |
| SHV-28, TEM-1-like | 0 | | 1 (2) | 1 (<1) |
| SHV-62 | 1 (2) | | 0 | 1 (<1) |
| SHV-71-like | 1 (2) | | 0 | 1 (<1) |
| TEM-1 | 0 | | 1 (2) | 1 (<1) |
| ESBLs | 8 (14) | | 6 (10) | 14 (12) |
| CTX-M-15 | 2 (4) | | 3 (5) | 5 (4) |
| CTX-M-15, OXA-1_OXA-30 | 5 (9) | | 2 (3) | 7 (6) |
| CTX-M-15, OXA-1_OXA-30-like | 1 (2) | | 1 (2) | 2 (2) |
| Plasmid AmpCs | 2 (4) | | 1 (2) | 3 (3) |
| CMY-42 | 0 | | 1 (2) | 1 (<1) |
| DHA-1 | 2 (4) | | 0 | 2 (2) |
| QRDR mutations | 5 (9) | | 7 (12) | 12 (11) |
| *gyrA* mutations | 5 (9) | | 7 (12) | 12 (11) |
| S83F, D87A | 4 (7) | | 5 (9) | 9 (8) |
| S83I | 0 | | 2 (3) | 2 (2) |
| S83Y, D87A | 1 (2) | | 0 | 1 (<1) |
| *parC* mutations | 5 (9) | | 7 (12) | 12 (11) |
| S80I | 5 (9) | | 7 (12) | 12 (11) |
| PMQR gene-positive | 13 (23) | | 12 (21) | 25 (22) |
| *aac(6’)-Ib-cr, oqxA, oqxB, qnrB1* | 1 (2) | | 0 | 1 (<1) |
| *aac(6’)-Ib-cr, oqxA, oqxB19, qnrB1* | 1 (2) | | 1 (2) | 2 (2) |
| *aac(6’)-Ib-cr, oqxA, oqxB20*-like | 3 (5) | | 2 (3) | 5 (4) |
| *aac(6’)-Ib-cr, oqxA, oqxB20-like, qnrB1* | 0 | | 1 (2) | 1 (<1) |
| *aac(6’)-Ib-cr, oqxA11, oqxB19, qnrB1* | 1 (2) | | 1 (2) | 2 (2) |
| *oqxA, oqxB, qnrB1* | 0 | | 1 (2) | 1 (<1) |
| *oqxA, oqxB*-like | 1 (2) | | 0 | 1 (<1) |
| *oqxA, oqxB19* | 0 | | 1 (2) | 1 (<1) |
| *oqxA, oqxB19, qnrB1* | 0 | | 1 (2) | 1 (<1) |
| *oqxA, oqxB19, qnrB4* | 1 (2) | | 0 | 1 (<1) |
| *oqxA, oqxB19, qnrS1* | 0 | | 1 (2) | 1 (<1) |
| *oqxA, oqxB20*-like | 1 (2) | | 1 (2) | 2 (2) |
| *oqxA, oqxB20*-like, *qnrS1* | 0 | | 1 (2) | 1 (<1) |
| *oqxA, oqxB25*-like, *qnrB19* | 0 | | 1 (2) | 1 (<1) |
| *oqxA*, *oqxB5*, *qnrS1* | 1 (2) | | 0 | 1 (<1) |
| *oqxA*-like, *oqxB5, qnrS1* | 1 (2) | | 0 | 1 (<1) |
| *oqxA10, oqxB5, qnrS1* | 1 (2) | | 0 | 1 (<1) |
| *qnrB4* | 1 (2) | | 0 | 1 (<1) |
| *qnrS1* | 0 | | 1 (2) | 1 (<1) |
| Epidemiological clones |  | |  |  |
| ST15 | 3 (5) | | 4 (7) | 7 (6) |
|  |  | |  |  |
| *Proteus mirabilis* | 34 (4) | | 33 (5) | 67 (5) |
| *ꞵ*-lactamase gene-positive | 2 (6) | | 0 | 2 (3) |
| Narrow-spectrum *ꞵ*-lactamases | 1 (3) | | 0 | 1 (1) |
| TEM-1 | 1 (3) | | 0 | 1 (1) |
| ESBLs | 2 (6) | | 0 | 2 (3) |
| CTX-M-27 | 1 (3) | | 0 | 1 (1) |
| CTX-M-65, OXA-1_OXA-30 | 1 (3) | | 0 | 1 (1) |
| QRDR mutations | 11 (32) | | 3 (9) | 14 (21) |
| *gyrA* mutations | 11 (32) | | 3 (9) | 14 (21) |
| S83I | 3 (9) | | 3 (9) | 6 (9) |
| S83I, E87G | 1 (3) | | 0 | 1 (1) |
| S83R | 7 (21) | | 0 | 7 (10) |
| *parC* mutations | 11 (32) | | 3 (9) | 14 (21) |
| S84I | 11 (32) | | 1 (3) | 12 (18) |
| S84R | 0 | | 2 (6) | 2 (3) |
| PMQR gene-positive | 1 (3) | | 0 | 1 (1) |
| *aac(6’)-Ib-cr* | 1 (3) | | 0 | 1 (1) |
|  |  | |  |  |
| *Enterobacter cloacae* complex | 6 (<1) | | 11 (2) | 17 (1) |
| *ꞵ*-lactamase gene-positive | 0 | | 3 (27) | 3 (18) |
| Plasmid AmpCs | 0 | | 3 (27) | 3 (18) |
| ACT-14 | 0 | | 2 (18) | 2 (12) |
| MIR-3 | 0 | | 1 (9) | 1 (6) |
|  |  | |  |  |
| *Citrobacter freundii* complex | 13 (2) | | 6 (<1) | 19 (1) |
| *ꞵ*-lactamase gene-positive | 6 (46) | | 0 | 6 (32) |
| ESBLs | 1 (8) | | 0 | 1 (5) |
| CTX-M-15 | 1 (8) | | 0 | 1 (5) |
| Plasmid AmpCs | 6 (46) | | 0 | 6 (32) |
| CFE-1-like | 1 (8) | | 0 | 1 (5) |
| CMY-48-like | 2 (15) | | 0 | 2 (11) |
| CMY-65-like | 1 (8) | | 0 | 1 (5) |
| CMY-66-like | 1 (8) | | 0 | 1 (5) |
| CMY-79-like | 1 (8) | | 0 | 1 (5) |
| QRDR mutations | 0 | | 1 (17) | 1 (5) |
| *gyrA* mutations | 0 | | 1 (17) | 1 (5) |
| T83I | 0 | | 1 (17) | 1 (5) |
| PMQR gene-positive | 1 (8) | | 0 | 1 (5) |
| *qnrB9* | 1 (8) | | 0 | 1 (5) |
|  |  | |  |  |
| *Citrobacter amalonaticus* group | 2 (<1) | | 0 | 2 (<1) |
| QRDR mutations | 1 (50) | | 0 | 1 (50) |
| *gyrA* mutations | 1 (50) | | 0 | 1 (50) |
| S83L, D87N | 1 (50) | | 0 | 1 (50) |
| *parC* mutations | 1 (50) | | 0 | 1 (50) |
| S80I | 1 (50) | | 0 | 1 (50) |
| *parE* mutations | 1 (50) | | 0 | 1 (50) |
| L416F | 1 (50) | | 0 | 1 (50) |
|  |  | |  |  |
| *Morganella morganii* | 8 (1) | | 5 (<1) | 13 (<1) |
| *ꞵ*-lactamase gene-positive | 4 (50) | | 0 | 4 (31) |
| Plasmid AmpCs | 4 (50) | | 0 | 4 (31) |
| DHA-1 | 2 (25) | | 0 | 2 (15) |
| DHA-17 | 1 (13) | | 0 | 1 (8) |
| DHA-22 | 1 (13) | | 0 | 1 (8) |
| QRDR mutations | 2 (25) | | 2 (40) | 4 (31) |
| *gyrA* mutations | 2 (25) | | 2 (40) | 4 (31) |
| S83I | 2 (25) | | 1 (20) | 3 (23) |
| S83R | 0 | | 1 (20) | 1 (8) |
| *parC* mutations | 2 (25) | | 2 (40) | 4 (31) |
| S84I | 2 (25) | | 2 (40) | 4 (31) |
| *parE* mutations | 0 | | 1 (20) | 1 (8) |
| D421N | 0 | | 1 (20) | 1 (8) |
| PMQR gene-positive | 1 (13) | | 2 (40) | 3 (23) |
| *aac(6’)-Ib-cr* | 1 (13) | | 1 (20) | 2 (15) |
| *qnrS1* | 0 | | 1 (20) | 1 (8) |
|  |  | |  |  |
| *Serratia marcescens* | 0 | | 3 (<1) | 3 (<1) |
| *ꞵ*-lactamase gene-positive | 0 | | 2 (67) | 2 (67) |
| Narrow-spectrum *ꞵ*-lactamases | 0 | | 2 (67) | 2 (67) |
| SRT-like | 0 | | 1 (33) | 1 (33) |
| TEM-1 | 0 | | 1 (33) | 1 (33) |
| ESBLs | 0 | | 1 (33) | 1 (33) |
| CTX-M-3 | 0 | | 1 (33) | 1 (33) |
| Carbapenemase | 0 | | 1 (33) | 1 (33) |
| NDM-1 | 0 | | 1 (33) | 1 (33) |
| Uncategorized *ꞵ*-lactamases | 0 | | 1 (33) | 1 (33) |
| SRT-2 | 0 | | 1 (33) | 1 (33) |
| QRDR mutations | 0 | | 1 (33) | 1 (33) |
| *gyrA* mutations | 0 | | 1 (33) | 1 (33) |
| S83I | 0 | | 1 (33) | 1 (33) |
| *parC* mutations | 0 | | 1 (33) | 1 (33) |
| S84I | 0 | | 1 (33) | 1 (33) |

*^a^*Percentage of each qualifying uropathogen was calculated using the total number of baseline qualifying uropathogens at baseline as the denominator. Percentage of each genotypic subcategory was calculated using the number of each respective baseline qualifying uropathogen (present at ≥10^5^ CFU/mL) as the denominator in a posthoc analysis. For MLSTs, O:H serotypes, and *fimH* alleles, percentages do not reflect prevalence among each species as only a selected subset of isolates were tested (based on predefined phenotypic/MIC criteria [Supplemental Material] and additionally all microbiological failure isolates were characterized by MLST). Uropathogens may have more than 1 genotype. Bacterial species are only shown for qualifying uropathogens with a sufficient number of genotypes to display; refer to the drug‑resistant phenotype co‑publication for these additional uropathogens (Scangarella-Oman NE et al, submitted for publication).

*^b^*Only a subset of isolates was selected for genotypic characterization based on predefined phenotypic criteria (Supplemental Material). Only genotypes with *n* ≥5 isolates for the pooled trials and both treatment groups combined are displayed for the MLST and O:H genotypic categories.

**Table S2** Therapeutic, clinical, and microbiological success at TOC by selected baseline qualifying uropathogens and genotypic subcategories with *n* ≥10 isolates for at least 1 treatment group for pooled EAGLE-2 and EAGLE-3 data (ME-TOC population)

| **Uropathogen**  **Genotype/Clone*^a^*** | **Therapeutic success (participant-level)*^b^*** | |  | **Clinical success (participant-level)*^b^*** | |  | **Microbiological success  (uropathogen-level)*^c^*** | |
| --- | --- | --- | --- | --- | --- | --- | --- | --- |
|  | **Gepotidacin N = 620*^d^* n/N1 (%)*^b^*** | **Nitrofurantoin N = 596*^d^* n/N1 (%)*^b^*** |  | **Gepotidacin N = 620*^d^* n/N1 (%)*^b^*** | **Nitrofurantoin N = 596*^d^* n/N1 (%)*^b^*** |  | **Gepotidacin N = 620*^d^* n/N1 (%)*^c^*** | **Nitrofurantoin N = 596*^d^* n/N1 (%)*^c^*** |
| *Escherichia coli* | 311/496 (62.7) | 236/481 (49.1) |  | 368/496 (74.2) | 331/481 (68.8) |  | 415/502 (82.7) | 330/486 (67.9) |
| Narrow-spectrum *ꞵ*-lactamases | 19/27 (70.4) | 9/24 (37.5) |  | 23/27 (85.2) | 14/24 (58.3) |  | 21/27 (77.8) | 15/24 (62.5) |
| TEM-1 | 8/14 (57.1) | 4/16 (25.0) |  | 10/14 (71.4) | 7/16 (43.8) |  | 10/14 (71.4) | 9/16 (56.3) |
| ESBLs | 44/69 (63.8) | 20/57 (35.1) |  | 54/69 (78.3) | 31/57 (54.4) |  | 56/69 (81.2) | 36/57 (63.2) |
| CTX-M-15 | 11/20 (55.0) | 8/14 (57.1) |  | 13/20 (65.0) | 9/14 (64.3) |  | 16/20 (80.0) | 10/14 (71.4) |
| CTX-M-15, OXA-1_OXA-30 | 11/19 (57.9) | 3/14 (21.4) |  | 14/19 (73.7) | 6/14 (42.9) |  | 15/19 (78.9) | 8/14 (57.1) |
| CTX-M-27 | 16/21 (76.2) | 7/17 (41.2) |  | 19/21 (90.5) | 10/17 (58.8) |  | 18/21 (85.7) | 12/17 (70.6) |
| Extended-spectrum AmpCs | 30/47 (63.8) | 14/37 (37.8) |  | 37/47 (78.7) | 20/37 (54.1) |  | 39/47 (83.0) | 25/37 (67.6) |
| Uncategorized-spectrum *ꞵ*-lactamases | 13/23 (56.5) | 12/29 (41.4) |  | 18/23 (78.3) | 18/29 (62.1) |  | 16/23 (69.6) | 19/29 (65.5) |
| QRDR mutations | 85/146 (58.2) | 56/135 (41.5) |  | 108/146 (74.0) | 86/135 (63.7) |  | 117/150 (78.0) | 82/136 (60.3) |
| *gyrA* mutations | 85/146 (58.2) | 56/135 (41.5) |  | 108/146 (74.0) | 86/135 (63.7) |  | 117/150 (78.0) | 82/136 (60.3) |
| S83L | 11/14 (78.6) | 5/10 (50.0) |  | 13/14 (92.9) | 8/10 (80.0) |  | 11/14 (78.6) | 6/10 (60.0) |
| S83L, D87N | 73/131 (55.7) | 49/124 (39.5) |  | 94/131 (71.8) | 77/124 (62.1) |  | 105/135 (77.8) | 74/124 (59.7) |
| *parC* mutations | 79/136 (58.1) | 51/127 (40.2) |  | 100/136 (73.5) | 79/127 (62.2) |  | 111/140 (79.3) | 76/127 (59.8) |
| S80I | 42/72 (58.3) | 34/76 (44.7) |  | 56/72 (77.8) | 54/76 (71.1) |  | 59/75 (78.7) | 44/76 (57.9) |
| S80I, E84V | 33/57 (57.9) | 17/49 (34.7) |  | 39/57 (68.4) | 25/49 (51.0) |  | 48/58 (82.8) | 32/49 (65.3) |
| *parE* mutations | 22/39 (56.4) | 17/41 (41.5) |  | 28/39 (71.8) | 30/41 (73.2) |  | 30/40 (75.0) | 20/41 (48.8) |
| L416F | 22/39 (56.4) | 17/41 (41.5) |  | 28/39 (71.8) | 30/41 (73.2) |  | 30/40 (75.0) | 20/41 (48.8) |
| PMQR gene-positive | 29/45 (64.4) | 6/35 (17.1) |  | 35/45 (77.8) | 15/35 (42.9) |  | 37/46 (80.4) | 17/35 (48.6) |
| *aac(6’)-Ib-cr* | 13/21 (61.9) | 4/18 (22.2) |  | 15/21 (71.4) | 8/18 (44.4) |  | 19/22 (86.4) | 10/18 (55.6) |
| *qnrS1* | 8/12 (66.7) | 2/4 (50.0) |  | 9/12 (75.0) | 3/4 (75.0) |  | 10/12 (83.3) | 3/4 (75.0) |
|  |  |  |  |  |  |  |  |  |
| *Klebsiella pneumoniae* | 21/48 (43.8) | 16/43 (37.2) |  | 25/48 (52.1) | 25/43 (58.1) |  | 38/50 (76.0) | 26/44 (59.1) |
| Narrow-spectrum *ꞵ*-lactamases | 5/10 (50.0) | 1/6 (16.7) |  | 6/10 (60.0) | 3/6 (50.0) |  | 7/10 (70.0) | 3/6 (50.0) |
| PMQR gene-positive | 5/11 (45.5) | 3/8 (37.5) |  | 6/11 (54.5) | 4/8 (50.0) |  | 8/12 (66.7) | 6/9 (66.7) |
|  |  |  |  |  |  |  |  |  |
| *Proteus mirabilis* | 24/34 (70.6) | 9/30 (30.0) |  | 27/34 (79.4) | 15/30 (50.0) |  | 32/34 (94.1) | 21/30 (70.0) |
| QRDR mutations | 7/11 (63.6) | 1/3 (33.3) |  | 9/11 (81.8) | 2/3 (66.7) |  | 9/11 (81.8) | 2/3 (66.7) |
| *gyrA* mutations | 7/11 (63.6) | 1/3 (33.3) |  | 9/11 (81.8) | 2/3 (66.7) |  | 9/11 (81.8) | 2/3 (66.7) |
| *parC* mutations | 7/11 (63.6) | 1/3 (33.3) |  | 9/11 (81.8) | 2/3 (66.7) |  | 9/11 (81.8) | 2/3 (66.7) |
| S84I | 7/11 (63.6) | 0/1 (0) |  | 9/11 (81.8) | 1/1 (100) |  | 9/11 (81.8) | 0/1 (0) |

The ME-TOC population was defined as participants who were included in the micro-ITT population plus followed important components of the trial (i.e., received at least 80% of planned doses as randomized, actual treatment received was the same as the randomized treatment, had an interpretable quantitative urine culture at TOC, had evaluable clinical scores at baseline and TOC, did not receive any other systemic antimicrobials before TOC unless it was taken for the current infection, and had no other major protocol deviation that prevented evaluation of efficacy).

*^a^*Only baseline qualifying uropathogens (present at ≥10^5^ CFU/mL) and genotypic subcategories with *n* ≥10 isolates for the pooled studies in at least 1 treatment group are displayed. Only a subset of isolates was selected for genotypic characterization based on predefined phenotypic criteria. Uropathogens may have more than 1 genotype.

*^b^*For clinical and therapeutic response, a participant was counted once under a uropathogen category if multiple qualifying uropathogens within that category were isolated at baseline for the participant. Participants for whom all uropathogens were not eradicated and all symptoms were not resolved were considered therapeutic failures for all uropathogens. For therapeutic and clinical success: n/N1=(n) the number of participants within the category with a response of success/(N1) the total number of participants within the category and is the denominator for corresponding percentages.

*^c^*For microbiological response, a participant was counted more than once under a uropathogen category if multiple qualifying uropathogens within that category were isolated at baseline for the participant. For microbiological success: n/N1=(n) the number of isolates that are a microbiological success/(N1) the total number of isolates in the category and is the denominator for the corresponding percentages.

*^d^*The N in the header represents the total number of participants in the treatment arm.

**Table S3** Therapeutic, clinical, and microbiological success at follow-up by selected baseline qualifying uropathogens and genotypic subcategories with *n* ≥10 isolates for at least 1 treatment group for pooled EAGLE-2 and EAGLE -3 data (ME-FU population)

| **Uropathogen**  **Genotype/Clone*^a^*** | **Therapeutic success (participant-level)*^b^*** | |  | **Clinical success (participant-level)*^b^*** | |  | **Microbiological success  (uropathogen-level)*^c^*** | |
| --- | --- | --- | --- | --- | --- | --- | --- | --- |
|  | **Gepotidacin N = 597*^d^* n/N1 (%)*^b^*** | **Nitrofurantoin N = 581*^d^* n/N1 (%)*^b^*** |  | **Gepotidacin N = 597*^d^* n/N1 (%)*^b^*** | **Nitrofurantoin N = 581*^d^* n/N1 (%)*^b^*** |  | **Gepotidacin N = 597*^d^* n/N1 (%)*^c^*** | **Nitrofurantoin N = 581*^d^* n/N1 (%)*^c^*** |
| *Escherichia coli* | 212/472 (44.9) | 174/474 (36.7) |  | 313/472 (66.3) | 292/474 (61.6) |  | 293/478 (61.3) | 242/479 (50.5) |
| Narrow-spectrum *ꞵ*-lactamases | 11/27 (40.7) | 8/28 (28.6) |  | 18/27 (66.7) | 14/28 (50.0) |  | 13/27 (48.1) | 12/28 (42.9) |
| TEM-1 | 5/15 (33.3) | 4/19 (21.1) |  | 8/15 (53.3) | 8/19 (42.1) |  | 6/15 (40.0) | 7/19 (36.8) |
| ESBLs | 28/66 (42.4) | 15/58 (25.9) |  | 44/66 (66.7) | 26/58 (44.8) |  | 40/66 (60.6) | 24/58 (41.1) |
| CTX-M-15 | 7/18 (38.9) | 6/16 (37.5) |  | 9/18 (50.0) | 8/16 (50.0) |  | 11/18 (61.1) | 7/16 (43.8) |
| CTX-M-15, OXA-1_OXA-30 | 6/17 (35.3) | 2/13 (15.4) |  | 12/17 (70.6) | 3/13 (23.1) |  | 10/17 (58.8) | 4/13 (30.8) |
| CTX-M-27 | 10/21 (47.6) | 6/18 (33.3) |  | 16/21 (76.2) | 10/18 (55.6) |  | 12/21 (57.1) | 11/18 (61.1) |
| Extended-spectrum AmpCs | 16/43 (37.2) | 9/38 (23.7) |  | 28/43 (65.1) | 17/38 (44.7) |  | 25/43 (58.1) | 15/38 (39.5) |
| Uncategorized-spectrum *ꞵ*-lactamases | 9/23 (39.1) | 9/27 (33.3) |  | 14/23 (60.9) | 14/27 (51.9) |  | 12/23 (52.2) | 14/27 (51.9) |
| QRDR mutations | 52/146 (35.6) | 48/138 (34.8) |  | 94/146 (64.4) | 80/138 (58.0) |  | 77/150 (51.3) | 65/139 (46.8) |
| *gyrA* mutations | 52/146 (35.6) | 48/138 (34.8) |  | 94/146 (64.4) | 80/138 (58.0) |  | 77/150 (51.3) | 65/139 (46.8) |
| S83L | 8/15 (53.3) | 3/10 (30.0) |  | 12/15 (80.0) | 6/10 (60.0) |  | 9/15 (60.0) | 4/10 (40.0) |
| S83L, D87N | 43/131 (32.8) | 43/127 (33.9) |  | 82/131 (62.6) | 73/127 (57.5) |  | 67/34 (50.0) | 59/127 (46.5) |
| *parC* mutations | 47/136 (34.6) | 45/130 (34.6) |  | 86/136 (63.2) | 75/130 (57.7) |  | 72/140 (51.4) | 61/130 (46.9) |
| S80I | 26/74 (35.1) | 31/78 (39.7) |  | 48/74 (64.9) | 55/78 (70.5) |  | 40/76 (52.6) | 36/78 (46.2) |
| S80I, E84V | 18/56 (32.1) | 14/49 (28.6) |  | 34/56 (60.7) | 20/49 (40.8) |  | 29/57 (50.9) | 25/49 (51.0) |
| *parE* mutations | 12/39 (30.8) | 17/43 (39.5) |  | 25/39 (64.1) | 30/43 (69.8) |  | 18/39 (46.2) | 19/43 (44.2) |
| L416F | 12/39 (30.8) | 17/43 (39.5) |  | 25/39 (64.1) | 30/43 (69.8) |  | 18/39 (46.2) | 19/43 (44.2) |
| PMQR gene-positive | 17/42 (40.5) | 4/33 (12.1) |  | 26/42 (61.9) | 10/33 (30.3) |  | 26/43 (60.5) | 8/33 (24.2) |
| *aac(6’)-Ib-cr* | 7/20 (35.0) | 2/16 (12.5) |  | 13/20 (65.0) | 4/16 (25.0) |  | 12/21 (57.1) | 4/16 (25.0) |
| *qnrS1* | 5/11 (45.5) | 2/5 (40.0) |  | 6/11 (54.52) | 2/5 (40.0) |  | 8/11 (72.7) | 2/5 (40.0) |
|  |  |  |  |  |  |  |  |  |
| *Klebsiella pneumoniae* | 17/47 (36.2) | 10/42 (23.8) |  | 22/47 (46.8) | 18/42 (42.9) |  | 34/49 (69.4) | 19/43 (44.2) |
| Narrow-spectrum *ꞵ*-lactamases | 3/10 (30.0) | 1/5 (20.0) |  | 6/10 (60.0) | 1/5 (20.0) |  | 5/10 (50.0) | 2/5 (40.0) |
| PMQR gene-positive | 3/11 (27.3) | 1/8 (12.5) |  | 6/11 (54.5) | 2/8 (25.0) |  | 6/12 (50.0) | 3/9 (33.3) |
|  |  |  |  |  |  |  |  |  |
| *Proteus mirabilis* | 22/34 (64.7) | 7/31 (22.6) |  | 26/34 (76.5) | 13/31 (41.9) |  | 32/34 (94.1) | 16/31 (51.6) |
| QRDR mutations | 6/11 (54.5) | 1/3 (33.3) |  | 9/11 (81.8) | 2/3 (66.7) |  | 9/11 (81.8) | 1/3 (33.3) |
| *gyrA* mutations | 6/11 (54.5) | 1/3 (33.3) |  | 9/11 (81.8) | 2/3 (66.7) |  | 9/11 (81.8) | 1/3 (33.3) |
| *parC* mutations | 6/11 (54.5) | 1/3 (33.3) |  | 9/11 (81.8) | 2/3 (66.7) |  | 9/11 (81.8) | 1/3 (33.3) |
| S84I | 6/11 (54.5) | 0/1 (0) |  | 9/11 (81.8) | 1/1 (100) |  | 9/11 (81.8) | 0/1 (0) |

The ME-FU population was defined similarly to the ME-TOC population (Table S2), except instead required that participants had an interpretable quantitative urine culture at FU (unless the microbiological outcome at TOC was persistence or recurrence) and evaluable clinical scores at baseline and FU (unless clinical outcome at TOC was clinical improvement or worsening).

*^a^*Only baseline qualifying uropathogens (present at ≥10^5^ CFU/mL) and genotypic subcategories with *n* ≥10 isolates for the pooled studies in at least 1 treatment group are displayed. Only a subset of isolates was selected for genotypic characterization based on predefined phenotypic criteria. Uropathogens may have more than 1 genotype.

*^b^*For clinical and therapeutic response, a participant was counted once under a uropathogen category if multiple qualifying uropathogens within that category were isolated at baseline for the participant. Participants for whom all uropathogens were not eradicated and all symptoms were not resolved were considered therapeutic failures for all uropathogens. For therapeutic and clinical success: n/N1=(n) the number of participants within the category with a response of success/(N1) the total number of participants within the category and is the denominator for corresponding percentages.

*^c^*For microbiological response, a participant was counted more than once under a uropathogen category if multiple qualifying uropathogens within that category were isolated at baseline for the participant. For microbiological success: n/N1=(n) the number of isolates that are a microbiological success/(N1) the total number of isolates in the category and is the denominator for the corresponding percentages.

*^d^*The N in the header represents the total number of participants in the treatment arm.

**Predefined criteria for molecular characterization for a subset of uropathogen isolates**

| **Isolates algorithm** | **Tests** |
| --- | --- |
| Enterobacterales isolates displaying MIC results of ≥2 µg/mL for ceftriaxone and/or ceftazidime and/or cefotaxime and/or aztreonam. Additionally, *P. mirabilis* isolates with cefpodoxime, ceftazidime, and cefotaxime MIC ≥2 µg/mL. | Screening of *ꞵ*-lactamase encoding genes (ESBL, non‑ESBL [such as narrow-spectrum *ꞵ*-lactamases, uncategorized], plasmid AmpC, and carbapenemase genes) |
| Enterobacterales isolates displaying MIC results of ≥2 µg/mL for ceftriaxone or meropenem and for which isolates have no β-lactamase | Expression for Amp*C* |
| Enterobacterales isolates displaying MIC results of ≥2 µg/mL for ceftriaxone or meropenem | MLST |
| *E. coli* isolates displaying MIC results of ≥2 µg/mL for ceftriaxone or meropenem | O:H serotyping |
| *E. coli* isolates displaying MIC results of ≥2 µg/mL for ceftriaxone or meropenem and for which isolates have confirmed MLST131 | *fimH* typing |
| Enterobacterales isolates displaying ciprofloxacin MIC of >0.06 µg/mL or levofloxacin MIC of >0.12 µg/mL | QRDR |
| Enterobacterales isolates displaying ciprofloxacin MIC of >0.06 µg/mL or levofloxacin MIC of >0.12 µg/mL | Fluoroquinolone-resistance genes (such as the *qnr* family [*qnrA*, *qnrB*, *qnrC*, and *qnrS*], *qepA*, *oqxAB*, and *aac(6’)‑lb‑cr*) |
| *E. coli*isolates displaying ciprofloxacin MIC of >0.06 µg/mL or levofloxacin MIC of >0.12 µg/mL | Expression of AcrAB |
| *E. coli* isolates displaying gepotidacin MICs of ≥8 µg/mL | Fluoroquinolone-resistance genes (such as the *qnr* family [*qnrA*, *qnrB*, *qnrC*, and *qnrS*], *qepA*, *oqxAB*, and *aac(6’)‑lb‑cr*), QRDR, expression of AcrAB, and MLST |
| Isolates from the same species collected from the same participant during multiple study visits | MLST or PFGE |
